# PNA-Modified Liposomes Improve the Delivery Efficacy of CAPIRI for the Synergistic Treatment of Colorectal Cancer

**DOI:** 10.3389/fphar.2022.893151

**Published:** 2022-06-15

**Authors:** Wenbin Diao, Ben Yang, Sipeng Sun, Anping Wang, Rongguan Kou, Qianyun Ge, Mengqi Shi, Bo Lian, Tongyi Sun, Jingliang Wu, Jingkun Bai, Meihua Qu, Yubing Wang, Wenjing Yu, Zhiqin Gao

**Affiliations:** ^1^ School of Life Science and Technology, Weifang Medical University, Weifang, China; ^2^ Shandong Universities Key Laboratory of Biopharmaceuticals, Weifang, China; ^3^ Translational Medical Center, Second People’s Hospital of Weifang, Weifang, China

**Keywords:** colorectal cancer, liposomes, peanut agglutinin, combination therapy, irinotecan, capecitabine

## Abstract

Tumor-associated antigen mucin 1 (MUC1) is highly expressed in colorectal cancer and is positively correlated with advanced stage at diagnosis and poor patient outcomes. The combination of irinotecan and capecitabine is standard chemotherapy for metastatic colorectal cancer and is known as XELIRI or CAPIRI, which significantly prolongs the progression-free survival and overall survival of colorectal cancer patients compared to a single drug alone. We previously reported that peanut agglutinin (PNA)-conjugated liposomes showed enhanced drug delivery efficiency to MUC1-positive liver cancer cells. In this study, we prepared irinotecan hydrochloride (IRI) and capecitabine (CAP)-coloaded liposomes modified by peanut agglutinin (IRI/CAP-PNA-Lips) to target MUC1-positive colorectal cancer. The results showed that IRI/CAP-PNA-Lips showed an enhanced ability to target MUC1-positive colorectal cancer cells compared to unmodified liposomes. Treatment with IRI/CAP-PNA-Lips also increased the proportion of apoptotic cells and inhibited the proliferation of colorectal cancer cells. The targeting specificity for tumor cells and the antitumor effects of PNA-modified liposomes were significantly increased in tumor-bearing mice with no severe cytotoxicity to normal tissues. These results suggest that PNA-modified liposomes could provide a new delivery strategy for the synergistic treatment of colorectal cancer with clinical chemotherapeutic agents.

## Introduction

The 2020 Global Cancer Report showed that the incidence of colorectal cancer ranks third among all cancers (Engstrand et al., 2018; Xu, Fan, et al., 2019; Sung et al., 2021). The main treatment for colorectal cancer is surgical resection combined with chemotherapy (Chau and Cunningham, 2002). The topoisomerase I inhibitor irinotecan (IRI) mainly relies on its active metabolite SN-38 to exert tumor-suppressive effects and is widely used in the treatment of colorectal and pancreatic cancers but with toxic effects of neutrophil reduction and diarrhea (de Man et al., 2018). IRI is often used in combination with 5-FU and its derivatives in the clinical treatment of cancer (Paulík et al., 2020). Studies have shown that capecitabine (CAP) is a precursor of 5-FU, and its combination with irinotecan (XELIRI or CAPIRI) is as effective as other synergistic treatments in colorectal cancer patients. The therapy is well tolerated with fewer toxic side effects and a higher safety profile at the same level of efficacy (Goldberg, 2005; Walko and Lindley, 2005; Lam et al., 2016). Due to the limitations of conventional chemotherapeutic drugs, such as poor targeting, short drug clearance half-life, low bioavailability, and poor solubility, there is much room to improve the efficacy of chemotherapy. Liposomes are ideal carriers to encapsulate chemotherapy drugs, not only to reduce drug toxicity and improve drug stability but also to enhance antitumor efficacy through surface modifications for active targeting and precise drug delivery (Harashima et al., 1999; Barenholz, 2012; Mitragotri et al., 2015).

Mucin 1 (MUC1) is a type of tumor-associated carbon antigen (TACA), which is a highly glycosylated mucin, and studies have shown that it is an important biomarker in colorectal cancer (Nabavinia et al., 2017; Gao et al., 2020). Altered expression of MUC1 glycosyltransferase results in a Thomsen–Friedenreich (TF) structure, which reduces the adhesion between tumor cells. Studies have shown that high expression of MUC1 promotes tumor cell proliferation and metastasis, and its expression is proportional to tumor malignancy and is associated with poor prognosis in patients (Li et al., 2019). Thus, using MUC1-specific ligands to modify liposomes could enable targeted delivery of drugs to MUC1-positive colorectal cancer tissue. Peanut agglutinin (PNA) is a homotetrameric plant agglutinin extracted from peanuts that can bind to a variety of disaccharides containing β-D-galactosyl-(1–3)-N-acetyl-D-galactosamine [Gal-β(1–3)GalNAc]. Studies have found that the core component of the TF structure is Gal-β(1–3)GalNAc, which is the core structure of MUC1 (Beack et al., 2017; Kumagai et al., 2019). Therefore, PNA-modified liposomes can actively target MUC1-positive colorectal cancers to deliver drugs centrally. Previously, we reported that PNA-modified liposomes showed enhanced drug delivery efficiency toward MUC1-positive liver cancer cells, which suggested the feasibility of using PNA-modified liposomes targeting MUC1 as a means to enhance its antitumor effects (Li, Diao, et al., 2020).

In this study, we prepared PNA-modified liposomes coloaded with IRICAP (IRI/CAP-PNA-Lips), achieved targeted codelivery of drugs, and improved the effectiveness of the anti-colorectal cancer effect. PNA-modified liposomes coloaded with drugs are important as a novel drug delivery strategy to enhance the efficacy of the clinical synergistic treatment of colorectal cancer.

## Materials and Methods

### Synthesis and Identification of DSPE-PEG2K-PNA

Ten milligrams of peanut agglutinin (PNA, Medicago, Uppsala, Sweden) was weighed and dissolved in 2 ml of pH 7.4 PBS, and 0.2 ml (5.0 eq.) of DSPE-PEG2K-NHS (Xi’an Rui Xi Biotechnology Corporation, Xi’an, China) was added to DMSO solution. The reaction solution was transferred to a dialysis bag (cutoff molecular weight 8,000–14,000 Da) after 4 h of reaction at room temperature and then dialyzed with pure water for 12 h. The dialysate was collected and freeze-dried to obtain DSPE-PEG2K-PNA.

SDS–polyacrylamide gel electrophoresis (SDS–PAGE) was used for the analysis of PNA and DSPE-PEG2K-PNA. The gel was stained with 0.25% Coomassie Brilliant Blue solution for 3 h and then decolorized with the decolorizing solution until the bands were clear. The successful linkage of PNA and DSPE-PEG2K was determined using infrared spectroscopy analysis.

### Analysis of the Optimal Synergistic Ratio of Irinotecan Hydrochloride (IRI) and Capecitabine (CAP) Using the Combination Index Method

The CI method was used to analyze the optimal synergistic ratio of irinotecan hydrochloride (IRI, Yuanye Biotechnology Corporation, Shanghai, China) and capecitabine (CAP, Ark Pharm, Chicago, United States) (Meng et al., 2015). Four colorectal cancer cell lines, Caco-2, HCT116, HT29, and SW620 (Cell Resource Center, Institute of Basic Medical Sciences, Chinese Academy of Medical Sciences, Beijing, China), were treated with different concentrations of IRI or CAP alone for 72 h to obtain the half-maximal inhibitory concentration (IC50) of IRI and CAP, respectively. The concentrations of the main drug, IRI, were determined as 1 μg/ml, 3 μg/ml, 9 μg/ml, 27 μg/ml, and 81 μg/ml, and the amounts of CAP were added at ratios of IRI:CAP equal to 8:1, 4:1, 3:1, 2:1, 1:1, 1:2, 1:3, 1:4, and 1:8 to obtain forty-five different concentrations and ratios of IRI and CAP mixture. Concentrations of IRI equal to 1 μg/ml, 3 μg/ml, 9 μg/ml, 27 μg/ml, and 81 μg/ml, and equivalent concentrations of CAP alone were used as controls. The four cell lines were treated with forty-five different concentration conditions for 72 h, and the IC50 values were calculated for the combination of the two drugs at different ratios. Six replicate wells were used in each group.

Based on the IC50 values, CI at different IRI/CAP ratios was calculated using the following equation:

$$CI=IC_{1}/IC_{m1}+IC_{2}/IC_{m2}.$$

IC_1_ and IC_2_ represent the IC50 values of IRI and CAP when the two drugs are combined to treat cells. IC_m1_ and IC_m2_ represent the IC50 values of IRI and CAP when they are treated alone (Guo et al., 2020). If the CI is greater than 1, it means that the two drugs have an antagonistic effect, less than 1 means that the two drugs have a synergistic effect, and equal to 1 means that the two drugs have an additive effect.

### Preparation of PNA-Modified Liposomes Coloaded With IRICAP

Blank liposomes (Lips) were prepared by a thin-film dispersion method (Gao et al., 2021). Lecithin and cholesterol (Avet Pharmaceutical Corporation, Shanghai, China) were dissolved in 4 ml of chloroform at a mass ratio of 4:1, and the organic solvent was removed by heating and evaporating in a rotary evaporator (Yarmato Technology & Trading Corporation, Shanghai, China) to form lipid films. Then, 70 mg/ml ammonium sulfate was hydrated and homogenized for particle size using an ultrasonic cell crusher (Sonics & Materials, United States). The liposome extruders were passed over 0.2 and 0.1 μm filter membranes, repeatedly extruded 10 times, and dialyzed with ddH_2_O for 6 h to obtain unmodified blank liposomes.

Liposomes coloaded with IRICAP (IRI/CAP-Lips) and PNA-modified liposomes coloaded with IRICAP (IRI/CAP-PNA-Lips) were prepared by thin-film dispersion combined with the ammonium sulfate gradient method (Abraham et al., 2005; Zhang and Yao, 2012). Capecitabine was dissolved in chloroform according to the mass ratio (phospholipid:CAP = 1.3:1) to obtain loaded capecitabine liposomes (CAP-Lips). Then, CAP-Lips were mixed with IRI according to the mass ratio (phospholipid:IRI = 10:1), and IRI/CAP-Lips were obtained after hydration and dialysis. DSPE-PEG2K-PNA was used according to the mass ratio (phospholipid:PNA = 10:1) in chloroform, and IRI/CAP-PNA-Lips were obtained based on the IRI/CAP-Lip preparation procedure.

### Characterization of PNA-Modified Liposomes Coloaded With IRICAP

The liposomes of each type were ultrasonically crushed for 10 min and then passed through 0.2 and 0.1 μm membrane filters ten times, diluted 10-fold with ddH_2_O, and prepared for use. The morphological observation was performed using transmission electron microscopy (HITACHI High-Tech Corporation, Japan) after phosphotungstic acid staining (Feng et al., 2019). Particle size and potential were determined using a Malvern particle size meter (Malvern Panalytical Corporation, UK) (Sarisozen et al., 2012).

The encapsulation efficiency (EE) and drug loading (DL) of the coloaded liposomes were measured by using high-performance liquid chromatography (HPLC, Thermo Fisher Scientific, Massachusetts, United States) (Wang et al., 2019). After 6.8 g of potassium dihydrogen phosphate was dissolved in 800 ml of water, 10 ml of triethylamine was added, and the pH was adjusted to 4.0 with phosphoric acid. Water was then added to 1,000 ml to configure the phosphate buffer with a mobile phase ratio of methanol:acetonitrile:phosphate buffer equal to 55:5:45. After diluting the liposomes 10-fold, 0.2% Triton X-100 was added to permeabilize the membrane for 15 min, and the absorption peaks of IRI and CAP were detected at 254 and 307 nm, respectively. The EE and DL of liposomes were calculated using the following equations:

Encapsulation efficiency (EE) % = Mass of the drug loaded/Total mass of the drug used × 100%

Drug loading (DL) % = Mass of the drug loaded/Total mass of liposomes × 100%

### Stability and Drug Release Analysis of PNA-Modified Liposomes Coloaded With IRICAP

Each type of liposome was stored in saline and 3% BSA at 4°C for 16 days. The changes in particle size and potential over 16 days were measured using a Malvern particle size meter.

The *in vitro* dialysis method was used for the drug release analysis of liposomes (Liu et al., 2017; Li, Hou, et al., 2020). For this, 2 ml of each type of liposome was placed in a dialysis bag (with molecular weight cutoff at 8,000–14,000 Da), placed in a conical flask containing 100 ml of phosphate-buffered saline (PBS), and incubated at 37°C on a shaker at 100 rpm. At each time point (15 min, 30 min, 1, 8, 12, and 24 h), samples (2 ml) were taken from the PBS and immediately replenished with an equal volume of fresh PBS. The levels of IRI and CAP in the samples and the cumulative drug release were analyzed by HPLC.

### Identification of MUC1-Positive Colorectal Cancer Cell Lines

Caco-2, HCT116, HT29, and SW620 cells were cultured overnight in 6-well plates (3 × 10^5^ cells/well). Total RNA that had been extracted by the TRIzol method was used as the template to produce cDNA by using an RT-PCR kit (Toyobo Life Science, Shanghai, China). Furthermore, the cDNA was used as a template for real-time quantitative PCR, using a program that was set to 95°C, 30 s→95°C, 5 s; 60°C, 10 s; and 72°C, 15 s (40 cycles). The Ct values of each group were recorded, and the relative expression of each group was calculated by the 2^ (-Delta Delta C(T)) method.

The four cell lines were cultured overnight in 6-well plates (3 × 10^5^ cells/well), and RIPA lysis was performed to extract cellular protein. Western blot analysis identified MUC1 expression in the four cell lines.

Furthermore, all four cell lines were inoculated into cell crawls (5×10^4^ cells/well) for 24 h, fixed in 4% tissue cell fixative for 10 min, permeabilized with 0.1% Triton X-100 for 10 min, blocked with 3% BSA for 2 h, incubated overnight at 4°C with MUC1-specific antibody (Cell Signaling Technology, Danvers, Massachusetts, United States), and further incubated with fluorescent secondary antibody for 1 h. DAPI was used to stain the nuclei. The four cell lines were then visualized with a confocal laser scanning microscope (CLSM, Leica Microscope Imaging System, Germany) to observe MUC1 expression. After each step of the aforementioned treatment, the cells were rinsed with PBS three times.

### Targeting Property Analysis of PNA-Modified Liposomes *In Vitro*

To study the cellular uptake of liposomes by cells *in vitro*, FITC and rhodamine (Rhb) were used to replace the CAP and IRI to prepare liposomes coloaded with FITCRhb (FITC/Rhb-Lips) and PNA-modified liposomes coloaded with FITCRhb (FITC/Rhb-PNA-Lips). Caco-2, HCT116, HT29, and SW620 cells were inoculated into cell crawls (5 × 10^4^ cells/well) overnight. The cells were incubated for 4 h with the addition of complete medium containing equal concentrations of FITC/Rhb, FITC/Rhb-Lips, and FITC/Rhb-PNA-Lips (Khan et al., 2020). We used 0.1% Triton X-100 to permeabilize the cell membrane and nuclear membrane. DAPI was utilized to stain the nucleus and an anti-fluorescence quencher as a sealant. Images were taken to observe the fluorescence distribution and intensity of different cells with CLSM. Each of the aforementioned steps was performed in the dark, and the cells were rinsed with PBS three times after the treatment (Xu et al., 2019).

### Cytotoxicity Analysis of PNA-Modified Liposomes Coloaded With IRICAP *In Vitro*

Caco-2, HCT116, HT29, and SW620 cells were inoculated into 96-well plates (1 × 10^4^ cells/well) overnight. The old medium was discarded, and Lips, IRI/CAP, IRI/CAP-Lips, and IRI/CAP-PNA-Lips were added to a 1.5-fold concentration gradient of IRI for 72 h. The old medium was replaced with a medium containing MTS and incubated for 15 min at 37°C. Enzyme-linked immunoassay (BIO-RAD, Hercules, California, United States) was used to detect optical density (OD) values. Inhibition of cell viability and IC50 values were calculated after 72 h of treatment with PNA-modified liposomes.

The four cell lines were treated with complete medium containing Lips, IRI/CAP, IRI/CAP-Lips, and IRI/CAP-PNA-Lips (C_IRI_ = 25 μg/ml, 50 μg/ml, 100 μg/ml) for 24 h. The MTS assay was used to analyze the inhibition of cell viability (Wang et al., 2015; Thapa et al., 2017).

$$Cell\;Viability\left(\%\right)=OD_{sample}-OD_{blank}/OD_{control}-OD_{blank}\times 100\%$$

### Analysis of the Targeting Property of PNA-Modified Liposomes Coloaded With IRICAP *In Vivo*

To study the targeting property of liposomes *in vivo*, fluorescent-labeled liposomes were prepared by using Cy7 (Meilun Biotechnology Corporation, Dalian, China) at a mass ratio of phospholipid:Cy7 of 50:1 instead of the drugs encapsulated into the hydrophilic phase of liposomes.

BALB/C-nu mice (male, 4–5 weeks old, 14 ± 2 g, Viton River Laboratory Animal Technology Corporation, Beijing, China) were selected. All experiments met the institution’s animal care standards and were approved by the Ethical Review Committee of Weifang Medical College. SW620 cells in the logarithmic growth phase were resuspended to 5 × 10^7^cells/mL with saline and subcutaneously injected with 0.2 ml of cell suspension to construct a subcutaneous tumor-bearing nude mouse model.

When the mean tumor volume reached 100 mm^3^, tumor-bearing nude mice were divided equally into three groups (*n* = 6) for targeted property analysis *in vivo* (Jiang et al., 2016). The distribution of fluorescence signals was observed at 1, 2, 4, 8, 12, 24, and 48 h after tail vein injection of Cy7, Cy7-Lips, and Cy7-PNA-Lips (2.5 mg/kg) by using a small animal imaging system *in vivo* (PerkinElmer Inc., United States) (Song et al., 2014; Xiong et al., 2017). Nude mice were euthanized 48 h after injection, and the heart, liver, spleen, lung, and kidney tumors were photographed to analyze the distribution of fluorescence in vital organs and tumor tissues.

### Analysis of the Antitumor Ability of PNA-Modified Liposomes Coloaded With IRICAP *In Vivo*

Tumor-bearing nude mice were separated equally into 4 groups (*n* = 6) for antitumor experiments *in vivo*. Normal saline, IRI/CAP, IRI/CAP-Lips, and IRI/CAP-PNA-Lips (C_IRI_ = 20 mg/kg) were injected intraperitoneally every 4 days starting from the first day of grouping, and the body weight and tumor volume of nude mice were measured every other day (Guichard et al., 2001). After 20 days of treatment, tumor-bearing nude mice were euthanized; vital organs and tumor tissues were taken, photographed, and weighed; and the tumor inhibition rate (TIR) was calculated for each treatment group. The tumor tissues were fixed, embedded, and sectioned, and Ki-67 staining was performed to analyze the inhibitory ability of different treatments on the proliferation of tumor cells.

$$Tumor\;volume\left(mm^{3}\right)=length\times width^{2}\times 0.5$$

$$Tumor\;inhibition\;rate\left(TIR\right)=1-V_{treatment\;group}/V_{control\;group}\times 100\%$$

### Systemic Toxicity Assessment of PNA-Modified Liposomes Coloaded With IRICAP

Heart, liver, spleen, lung, and kidney tumors from the euthanized tumor-bearing nude mice were fixed, paraffin-embedded, and sectioned. H&E staining was performed on the tissue sections, and the sections were observed under a 20× microscope to determine whether obvious organic lesions appeared in each vital organ.

Heart blood was drawn from the SW620 tumor-bearing nude mouse model after 20 days of treatment, and the plasma was centrifuged for serum biochemical parameter analysis (Yu et al., 2019).

## Results

### Synthesis and Characterization of DSPE-PEG2K-PNA

**FIGURE 1:**
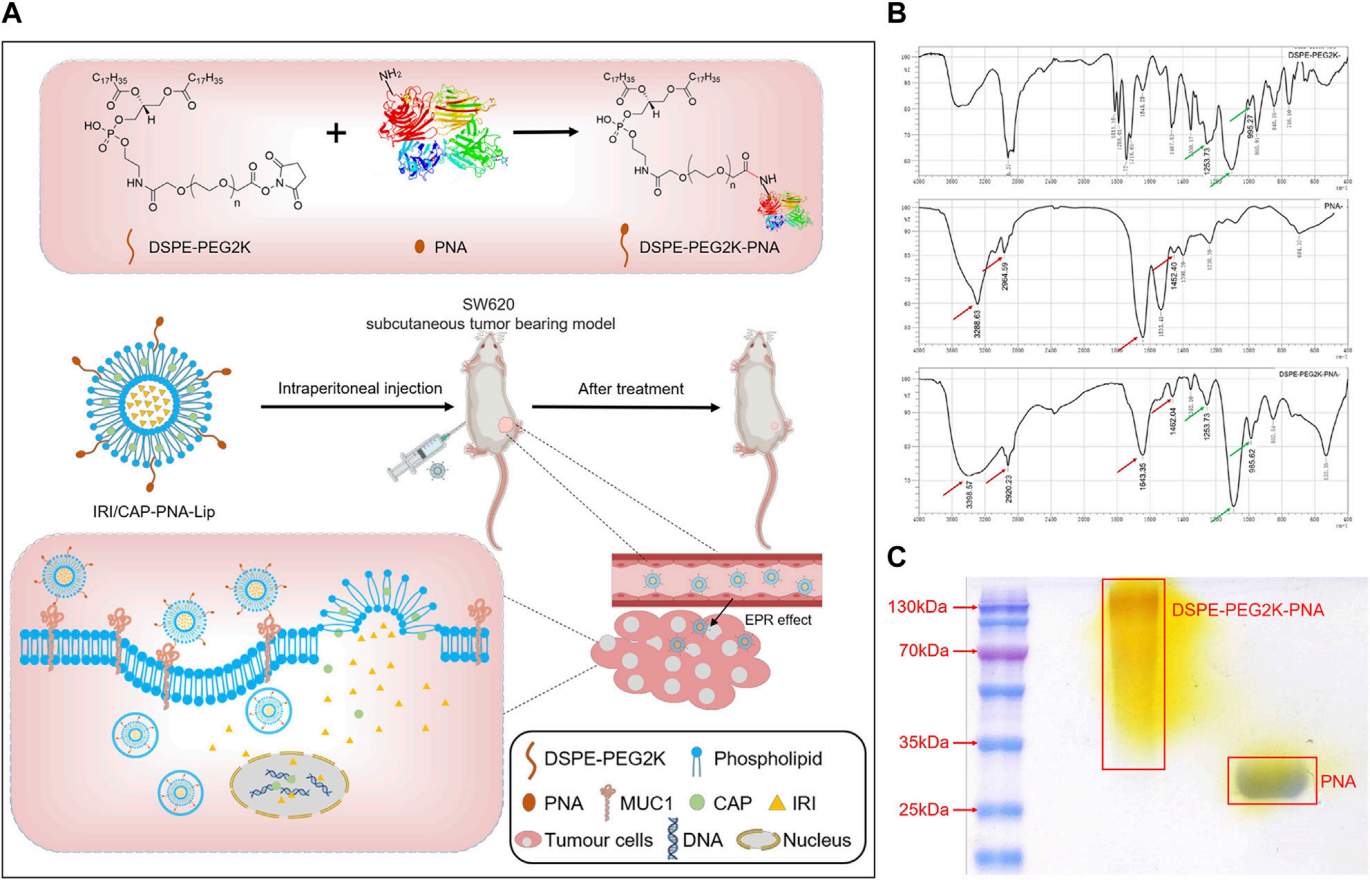
**(A)** Schematic diagram of the preparation of PNA-modified liposomes and ligand–receptor-mediated targeting mechanisms for tumor tissue recognition. Liposomes loaded with IRICAP were modified with PNA as the ligand. PNA specifically interacts with MUC1, which is highly expressed in colorectal cancer cells. IRI and CAP are released into cells and bind to DNA to inhibit nucleic acid synthesis, thus enhancing the efficacy of anti-colorectal cancer. **(B)** Infrared spectroscopy identified the successful synthesis of DSPE-PEG2K-PNA. **(C)** SDS–PAGE to identify PNA was ligated to DSPE-PEG2K.

DSPE-PEG2K was bonded to peanut agglutinin (PNA) through an amide bond (-CO-NH-). The presence of the characteristic peaks of both DSPE-PEG2K and PNA in the infrared spectroscopy (IR) spectrum of DSPE-PEG2K-PNA (Figure 1B) indicated that DSPE-PEG2K-PNA was successfully synthesized. The SDS–PAGE results also indicated that PNA was ligated to DSPE-PEG2K. PNA is a homotetramer with a molecular weight of approximately 120 kDa and a monomeric molecular weight of approximately 32 kDa, which was consistent with the band on the SDS–PAGE gel. The band of DSPE-PEG2K-PNA showed that the molecular weight was heavier than the molecular weight of PNA. In addition, PEG-specific staining clearly marked the DSPE-PEG2K-PNA band in yellow, while the PNA band had no color. These results suggest that we successfully synthesized DSPE-PEG2K-PNA (Figure 1C; Supplementary Figure S1D).

### The Optimal Synergistic Ratio of Irinotecan Hydrochloride and Capecitabine Is 3:1

**FIGURE 2:**
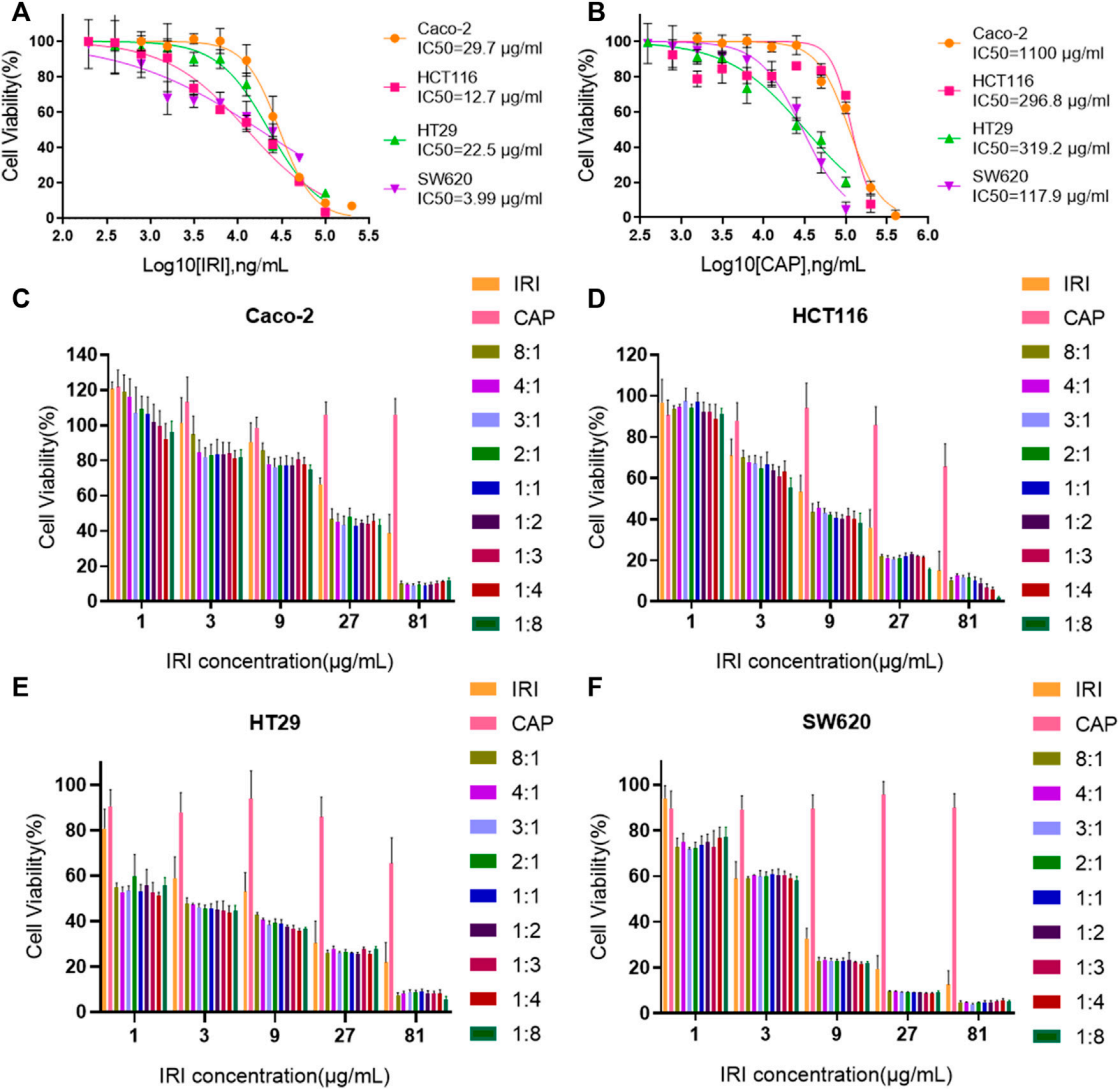
The optimal synergistic ratio of irinotecan hydrochloride and capecitabine was 3:1. **(A,B)** IC50 value analysis of CAP and IRI alone in Caco-2, HCT116, HT29, and SW620 cell lines. **(C–F)** Effect of IRI and CAP on the viability of Caco-2, HCT116, HT29, and SW620 cells at different ratios.

The ratio of irinotecan hydrochloride (IRI) and capecitabine (CAP) in clinical synergistic treatment is approximately 1:8 (Kerr, 2002). To evaluate the optimal synergistic ratio of IRI and CAP in the four colorectal cancer cell lines *in vitro*, the cell viability assay was performed by the MTS assay, and the combination index (CI) was compared (Figure 2). The CI of the four cell lines at different ratios was less than 1, suggesting that the combination of IRI and CAP exerted a synergistic effect. Caco-2, HT29, and SW620 had the lowest CI values when IRI:CAP was 3:1. The low CI values of HCT116 were 0.52 and 0.58 at IRI:CAP of 1:8 and 3:1, respectively (Table 1). The lowest CI values for Caco-2, HT29, and SW620 cells were observed at IRI:CAP of 3:1, and the lowest CI values were observed in HCT116 cells. We determined this ratio as the optimal synergistic ratio of the two drugs.

**TABLE 1:** CI values for four colorectal cancer cell lines at different combination ratios of IRI/CAP.

	Caco-2			HCT116			HT29			SW620		
	IC50 (IRI)	IC50 (CAP)	CI	IC50 (IRI)	IC50 (CAP)	CI	IC50 (IRI)	IC50 (CAP)	CI	IC50 (IRI)	IC50 (CAP)	CI
IRI	29.69	−	−	12.6	−	−	22.5	−	−	9.35	−	−
CAP	−	1100	−	−	296.8	−	−	319.2	−	−	117.9	−
8:01	26.3	3.29	0.89	7.44	0.93	0.59	5.01	0.63	0.22	3.2	0.4	0.35
4:01	22.1	5.53	0.75	7.57	1.9	0.61	5.11	1.28	0.23	2.9	0.725	0.32
3:01	20.1	6.7	0.68	7.22	2.41	0.58	4.89	1.63	0.22	2.7	0.9	0.29
2:01	22.2	11.1	0.76	6.78	3.39	0.55	4.98	2.49	0.22	3.1	1.55	0.34
1:01	20.4	20.4	0.71	6.94	6.94	0.57	5.08	5.08	0.24	2.7	2.7	0.31
1:02	20.6	41.2	0.73	6.44	12.88	0.55	5.12	10.24	0.26	2.7	5.4	0.33
1:03	21.4	64.2	0.78	6.17	18.51	0.55	4.96	14.88	0.26	2.5	7.5	0.33
1:04	20.6	82.4	0.77	6.03	24.12	0.56	4.89	19.56	0.27	2.3	9.2	0.32
1:08	19.5	156	0.8	4.9	39.2	0.52	4.86	38.88	0.33	2.6	20.8	0.45

### Characterization of PNA-Modified Liposomes Coloaded With CAPIRI

**FIGURE 3:**
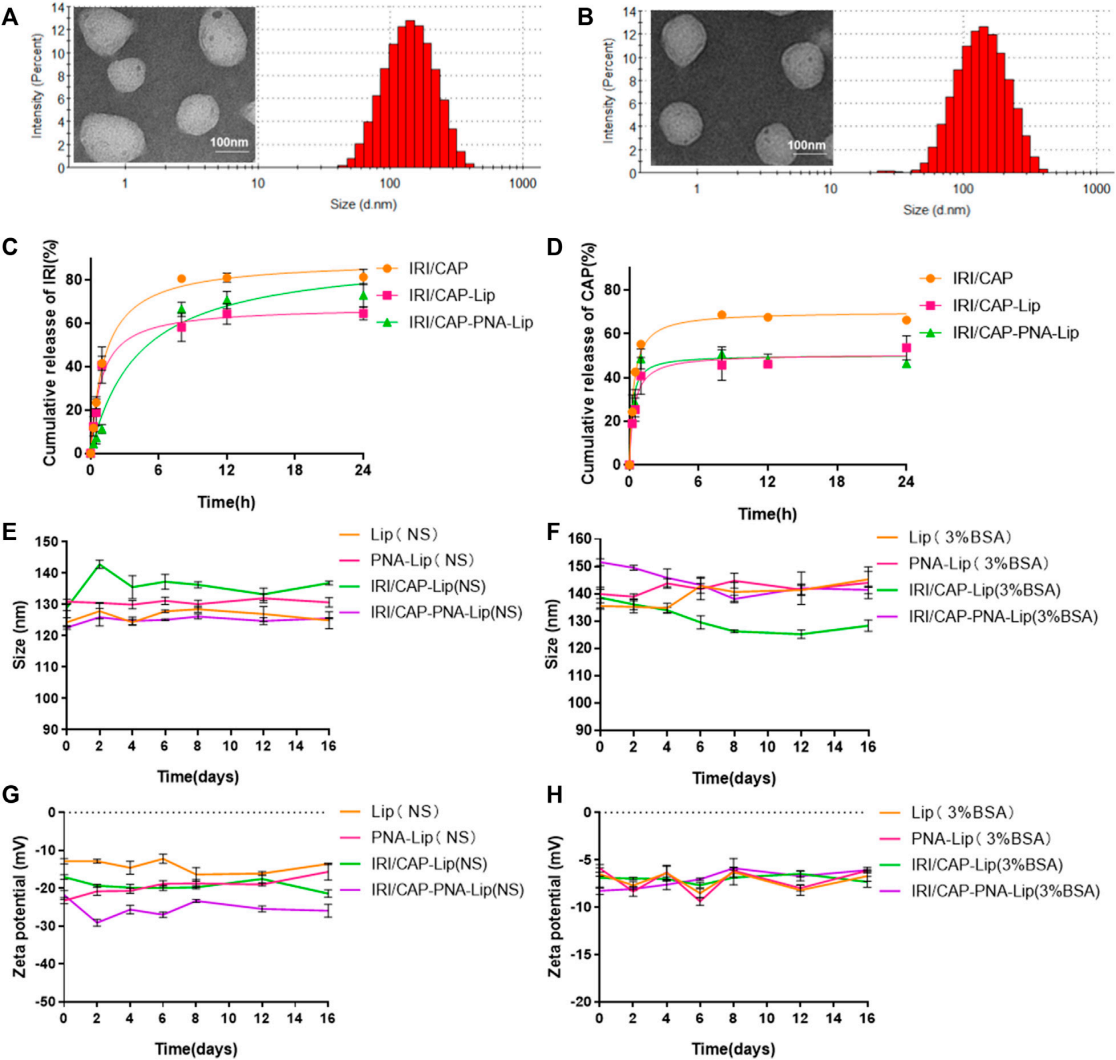
Analysis of the drug release capacity and stability of different types of drug-loaded liposomes. **(A,B)** Particle size histograms and transmission electron microscopy (TEMik) images of IRI/CAP-Lips and IRI/CAP-PNA-Lips. **(C)** Cumulative drug release profiles of IRI-loaded liposomes in PBS at 37°C. **(D)** Cumulative release curves of CAP-loaded liposomes in PBS at 37°C. **(E,F)** Folding graphs of particle size changes of different types of liposomes stored in saline or 3% BSA at 4°C for 16 days. **(G,H)** Folding graphs of potential changes of different types of liposomes stored in saline or 3% BSA at 4°C for 16 days. The data are presented as the mean ± SD (*n* = 3).

The particle size of PNA-modified liposomes coloaded with IRICAP (IRI/CAP-PNA-Lips) was 122.6 ± 0.58 nm. The polymer dispersity index (PDI) of coloaded PNA-modified liposomes was less than 0.2, which indicated that the particle size was dispersed equally (Figures 3A,B). For IRI/CAP-PNA-Lips, the encapsulation efficiency (EE) of IRI and CAP was 89.45 ± 0.015% and 7.56 ± 0.014%, respectively. The drug load (DL) of IRI and CAP was 17.98 ± 0.003% and 6.18 ± 0.013%, respectively (Table 2; Supplementary Figures S1A–C). The DL ratio of IRI and CAP in the coloaded PNA-modified liposomes was 3:1, which was consistent with the optimal synergistic ratio.

**TABLE 2:** Characteristics of different types of liposomes (mean ± SD, *n* = 3).

	Size (nm)	PDI	Zeta (mV)	EE IRI (%)	EE CAP (%)	DL IRI (%)	DL CAP (%)
Lips	126.6 ± 1.58	0.16 ± 0.04	−12.9 ± 0.70	−	−	−	−
PNA-Lips	130.9 ± 0.80	0.12 ± 0.02	−23.3 ± 0.81	−	−	−	−
IRI/CAP-Lips	126.7 ± 2.51	0.18 ± 0.05	−15.5 ± 0.56	57.67 ± 0.090	4.63 ± 0.020	11.53 ± 0.020	3.79 ± 0.010
IRI/CAP-PNA-Lips	122.6 ± 0.58	0.17 ± 0.03	−21.3 ± 0.49	89.45 ± 0.015	7.56 ± 0.014	17.98 ± 0.003	6.18 ± 0.013

A drug release assay was performed *in vitro* to evaluate the sustained release effect and maximum cumulative release of coloaded liposomes. The maximum cumulative release of free drugs was achieved at 8 h, with over 80% of IRI and 60% of CAP. The cumulative release curves of IRI and CAP in coloaded liposomes can be divided into two phases: initial rapid release and sustained release. IRI in IRI/CAP-PNA-Lips and IRI/CAP-Lips had a rapid release in the first 8 h, and the rapid release phase of CAP in IRI/CAP-PNA-Lips and IRI/CAP-Lip was reached in the first hour. After that, the curve gradually changed to a sustained release phase. Finally, the maximum cumulative release of IRI was reached at 24 h in IRI/CAP-PNA-Lips and IRI/CAP-Lips, with maximum release rates of 72.9 ± 5.3% and 64.6 ± 2.9%, respectively. The maximum cumulative release of CAP in IRI/CAP-PNA-Lips and IRI/CAP-Lips had maximum release rates of 50.7 ± 3.2% and 53.6 ± 5.5%, respectively (Figures 3C,D). The particle size and potential of each type of liposome were stable, and no significant changes were observed after storage in physiological saline or 3% BSA for half a month at 4°C (Figures 3E–H).

### MUC1 Was Highly Expressed in the Colorectal Cancer Cell Lines HT29 and SW620

**FIGURE 4:**
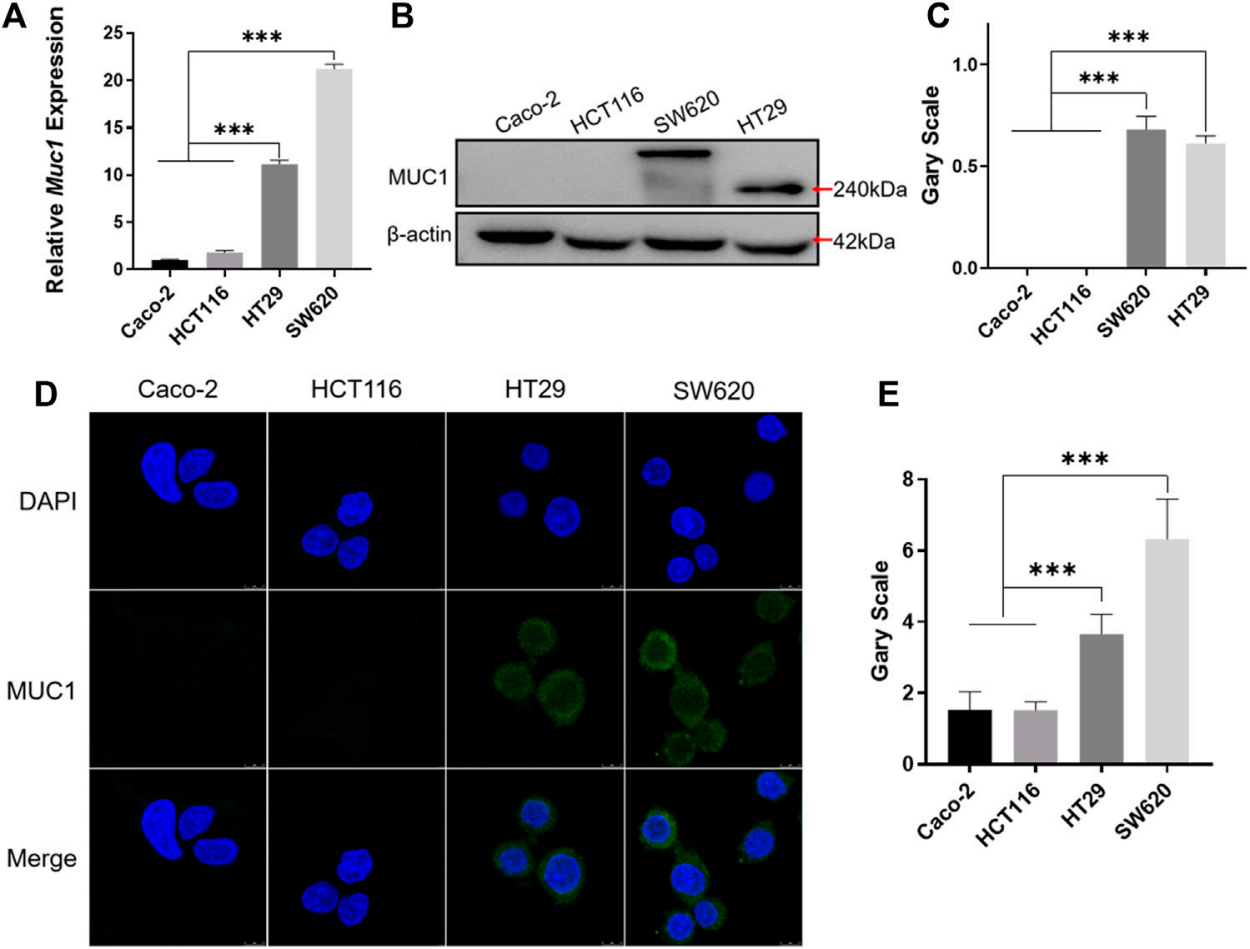
Identification of MUC1 expression in colorectal cancer cells. **(A)** Real-time quantitative PCR analysis of MUC1 expression at the mRNA level in Caco-2, HCT116, HT29, and SW620 cell lines. **(B)** Western blot analysis of MUC1 expression in four cell lines. **(C)** Quantitative histogram of Western blot results. **(D)** Cellular immunofluorescence assay for the identification of MUC1 expression in four cell lines. **(E)** Quantitative histogram of cellular immunofluorescence results. ***p < 0.001.

The results of real-time quantitative PCR and Western blotting showed that MUC1 was highly expressed in HT29 and SW620 cells (Figures 4A–C). Furthermore, immunofluorescence using a MUC1-specific antibody showed that the green fluorescence intensity was significantly higher in HT29 and SW620 cells than in Caco-2 and HCT116 cells (Figures 4D,E). The experiments showed that MUC1 was not expressed in the colorectal cancer cell lines Caco-2 and HCT116, while it was highly expressed in the colorectal cancer cell lines HT29 and SW620.

### Enhanced Cellular Uptake of PNA-Modified Liposomes by MUC1-Positive Colorectal Cancer Cell Lines

**FIGURE 5:**
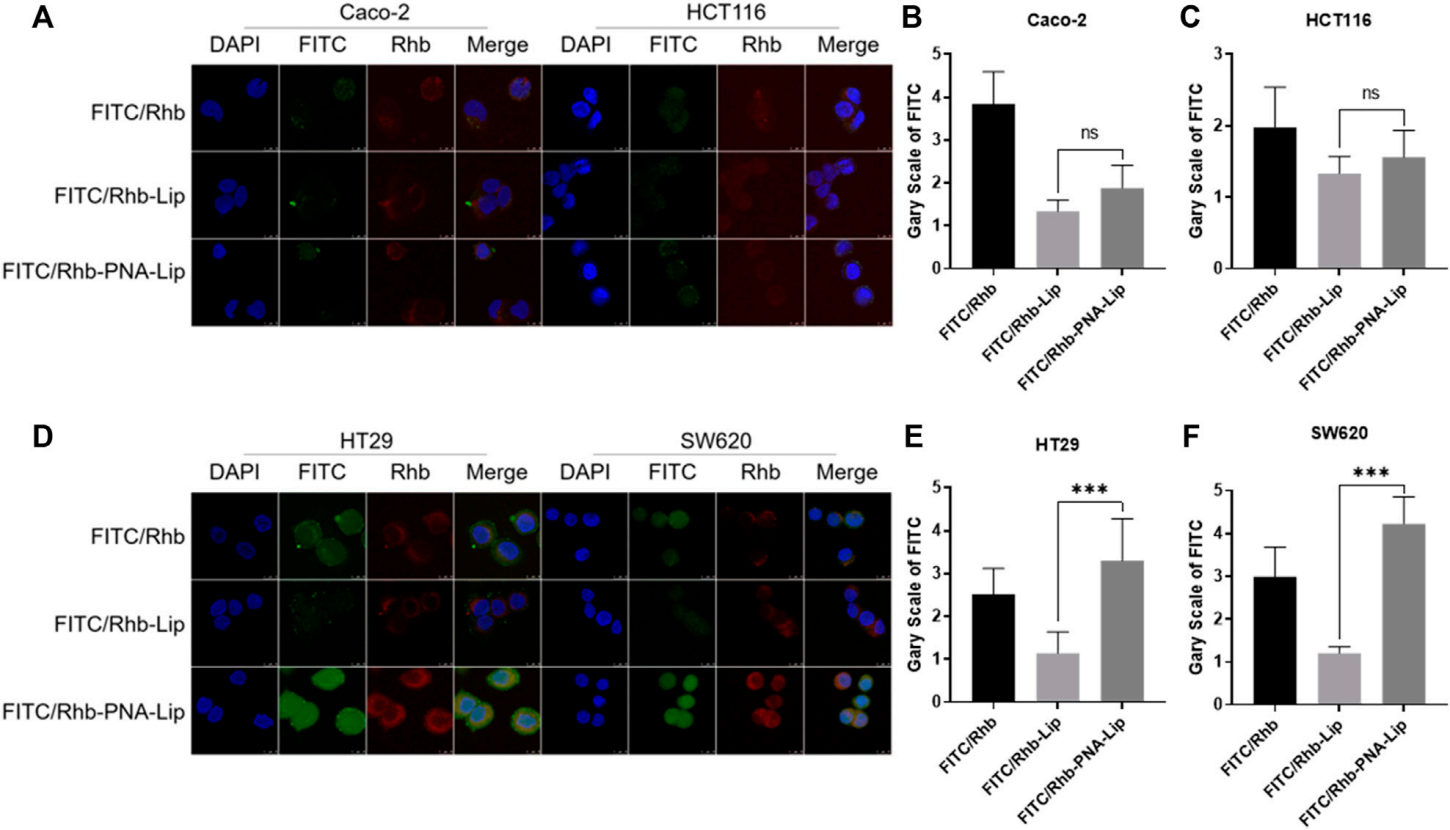
CLSM analysis showed that PNA-modified liposomes enhance the cellular uptake of drugs by MUC1-positive cells. **(A)** CLSM observation of the distribution of fluorescence signals in Caco-2 and HCT116 cells. **(B,C)** Quantitative histograms of FITC fluorescence signals in Caco-2 and HCT116 cells. **(D)** CLSM observation of the distribution of fluorescence signals in HT29 and SW620 cells. **(E,F)** Quantitative histograms of FITC fluorescence signals in HT29 and SW620 cells. The data are presented as the mean ± SD (n = 3). ***p < 0.001.

To explore the targeting efficiency of PNA-modified liposomes to MUC1-positive cells, PNA-modified liposomes loaded with FITC and rhodamine (Rhb) were prepared. The cellular uptake of FITC and Rhb in the four cell lines showed distinct results. Two MUC1-negative cell lines, Caco-2 and HCT116, showed no significant difference in the cellular uptake of FITC and Rhb in the FITC/Rhb-PNA-Lip groups compared to the FITC/Rhb-Lip groups. The fluorescence signals of the FITC/Rhb-PNA-Lip groups were significantly enhanced in HT29 and SW620 cells, which are MUC1-positive cell lines (Figure 5; Supplementary Figures S2A–D). The aforementioned results suggest that PNA-modified liposomes exhibit effective MUC1-targeting properties in colorectal cancer cells and could work as efficient drug delivery carriers to MUC1-positive colorectal cancer.

### PNA-Modified Liposomes Coloaded With IRICAP Significantly Inhibited the Viability of MUC1-Positive Colorectal Cancer Cell Lines *In Vitro*

**FIGURE 6:**
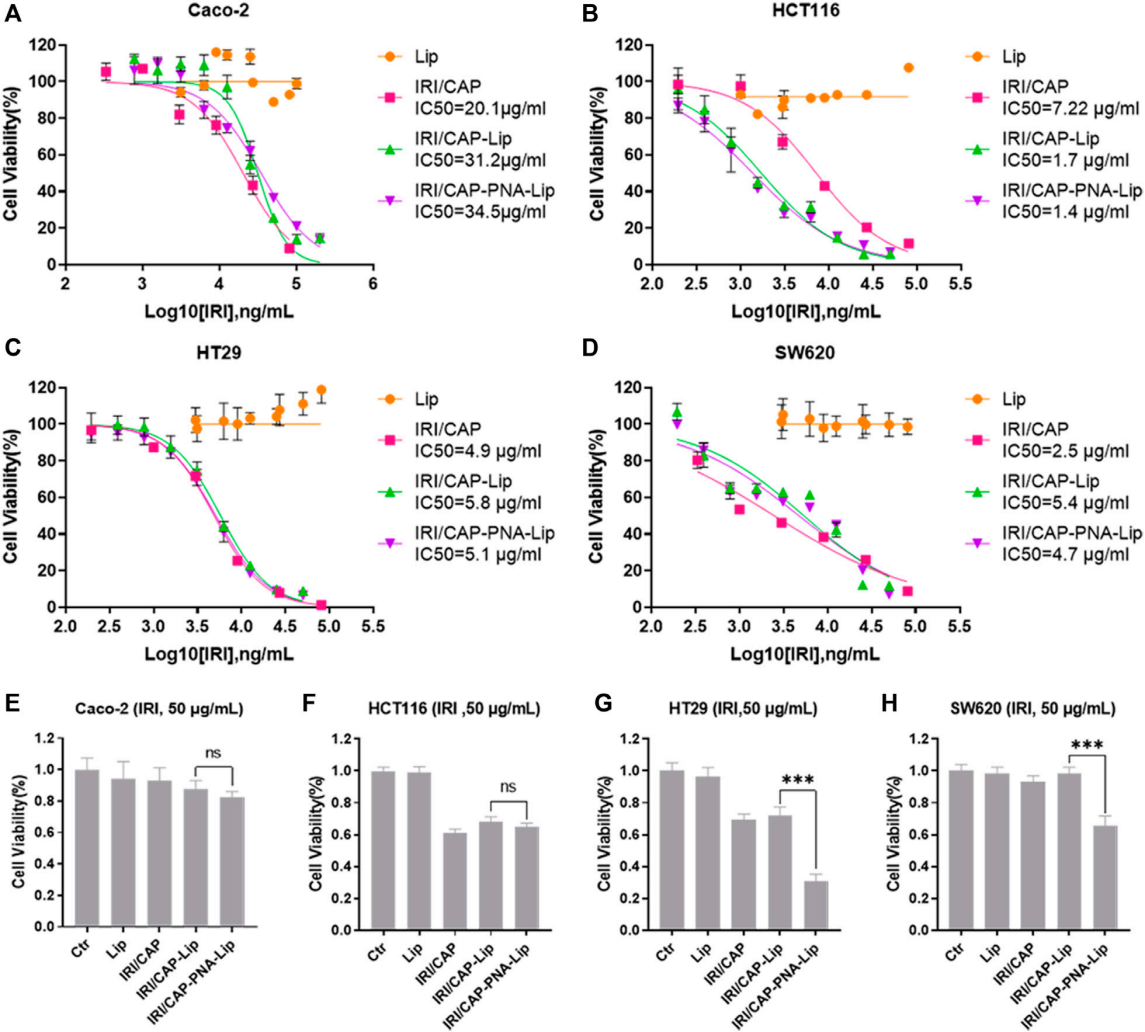
IRI/CAP-PNA-Lips significantly inhibited the viability of MUC1-positive colorectal cancer cell lines *in vitro*. **(A–D)** Cytotoxicity analysis of coloaded liposomes in Caco-2, HCT116, HT29, and SW620 cell lines. **(E–H)** Effect of an IRI concentration of 50 μg/ml on cell viability after treatment of Caco-2, HCT116, HT29, and SW620 cell lines for 24 h. The data are presented as the mean ± SD (*n* = 3). ***p < 0.001.

Based on the effectiveness of cellular uptake, the MTS assay was used to further evaluate the inhibitory effect of PNA-modified liposomes coloaded with IRICAP on the viability of colorectal cancer cell lines. The results showed that in the MUC1-positive colorectal cancer cell line HT29, the IC50 values for the IRI/CAP-Lip and IRI/CAP-PNA-Lip groups were 5.8 μg/ml and 5.1 μg/ml, respectively. Another MUC1-positive colorectal cancer cell line, SW620, had IC50 values for the IRI/CAP-Lip and IRI/CAP-PNA-Lip groups of 5.4 μg/ml and 4.7 μg/ml, respectively. There was no significant difference in the cytotoxicity of the IRI/CAP-Lip and IRI/CAP-PNA-Lip treatment groups (Figures 6A–D).

This result may be due to the complete cellular uptake of the liposomes coloaded with CAPIRI by all cells after 72 h. Therefore, HT29 and SW620 cells were treated individually for 24 h, and we discovered that IRI/CAP-PNA-Lips had a stronger inhibitory effect at IRI concentrations up to 50 μg/ml (Supplementary Figures S3A,B).

When a single concentration of coloaded liposomes (C_IRI_ = 50 μg/ml) was used to treat cells for 24 h, the viability of HT29 and SW620 cells was significantly inhibited by PNA-modified liposomes coloaded with IRICAP compared to unmodified liposomes, which was consistent with the result of 100 μg/ml IRI in coloaded liposomes (Figures 6E–H; Supplementary Figures S3C–J).

### PNA-Modified Liposomes Coloaded With IRICAP Promote Apoptosis in MUC1-Positive Colorectal Cancer Cells *In Vitro*

**FIGURE 7:**
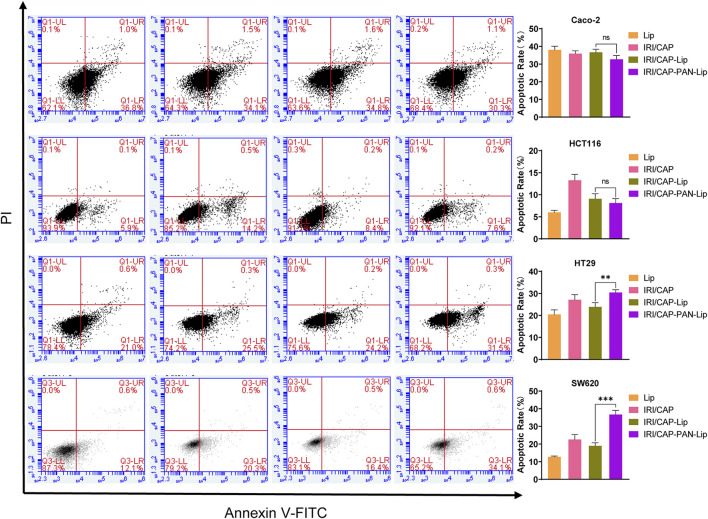
IRI/CAP-PNA-Lips significantly induced apoptosis compared to IRI/CAP-Lips in HT29 and SW620 cell lines. The effects of Lips, IRI/CAP, IRI/CAP-Lips, and IRI/CAP-PNA-Lips on the apoptosis of colorectal cancer cell lines and their quantitative histograms. The data are presented as the mean ± SD (*n* = 3). **p < 0.01. ***p < 0.001.

Based on the results of the MTS assays, we treated Caco-2, HCT116, HT29, and SW620 cells with coloaded liposomes at an IRI concentration of 50 μg/ml for 24 h. The effect of coloaded liposomes on the apoptosis of cells was evaluated using the Annexin V/PI double-staining method. The effects of IRI/CAP-Lips and IRI/CAP-PNA-Lips on the apoptosis of MUC1-negative Caco-2 and HCT116 cells were not significant. For MUC1-positive HT29 and SW620 cells, the apoptosis rate in the IRI/CAP-PNA-Lip group was 1.3-fold and 1.9-fold higher than that in the IRI/CAP-Lip group, respectively. The results suggested that PNA-modified liposomes coloaded with IRICAP significantly upregulated the apoptosis in MUC1-positive colorectal cancer cell lines (Figure 7).

### PNA-Modified Liposomes Significantly Increase MUC1-Targeting Properties in Colorectal Cancer Tumors *In Vivo*

Small animal live-imaging techniques were used to observe the distribution of Cy7 in tumor uptake to assess the targeting ability of PNA-modified liposomes *in vivo*. Fluorescence signals could be observed in the whole body 1 h after tail vein injection of Cy7, which barely disappeared after 12 h in the tumor tissue. For the Cy7-Lip and Cy7-PNA-Lip groups, fluorescence signals were observed in the whole body between 1 and 2 h after injection. However, there was slower decay of fluorescence signals in the tumor tissue, which was different from the free drug conditions. After 2 h, the fluorescence signals of the Cy7-Lip and Cy7-PNA-Lip groups were consistently stronger than those of the Cy7 group, and the fluorescence signal of the Cy7-PNA-Lip group was consistently stronger than that of the Cy7-Lip group.

**FIGURE 8:**
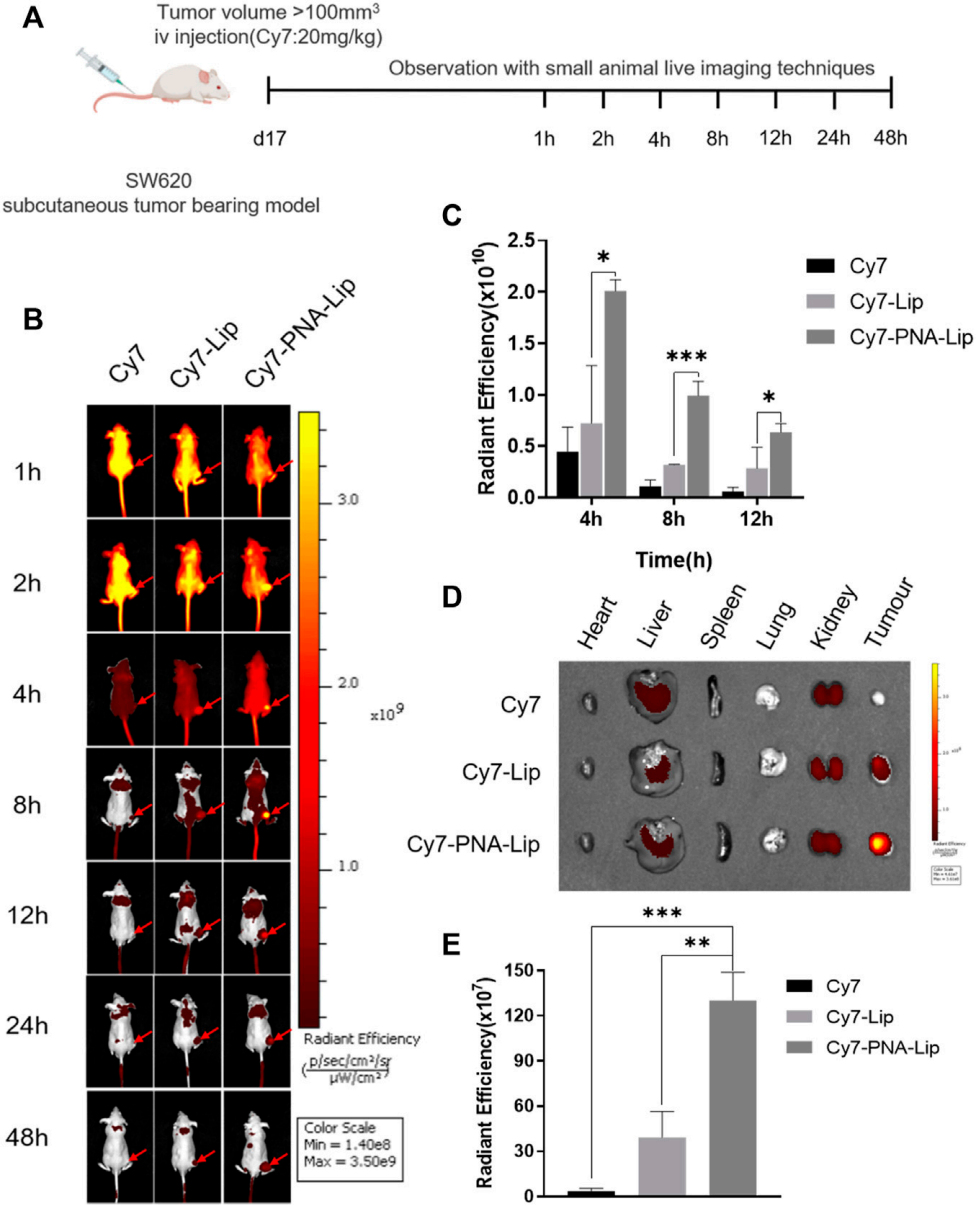
PNA-modified liposomes exhibited effective MUC1-targeting properties in colorectal cancer tumors. **(A)** Design principles for the analysis of the targeting ability of PNA-modified liposomes *in vivo*. **(B)** Distribution of Cy7 in tumor-bearing nude mice after tail vein injection. **(C)** Quantitative histogram of fluorescence signals at tumor sites at different points in time. **(D)** Distribution of Cy7 in vital organs and tumor tissues after 48 h. **(E)** Quantitative histograms of fluorescence signals in tumors. The data are presented as the mean ± SD (n = 3). *p < 0.05. **p < 0.01. ***p < 0.001.

The Cy7-PNA-Lip group showed the strongest fluorescence signals in the tumor tissue at 4 h after injection, which persisted in the tumor tissues up to 48 h after injection (Figure 8B). To verify that Cy7-PNA-Lips could improve the accumulation of drugs in tumor tissues and the targeted delivery efficiency, tumor-bearing nude mice were euthanized 48 h after injection (Figure 8C; Supplementary Figure S2E). Fluorescence accumulation was observed in the primary organs and tumor tissues. The Cy7-PNA-Lip group showed the strongest signals (Figure 8D). The results were consistent with the trend observed with cellular uptake, which suggested that PNA-modified liposomes could deliver targets and improve cellular uptake to improve drug accumulation in tumor tissues.

### PNA-Modified Liposomes Coloaded With IRICAP Have Shown Improved Antitumor Ability *In Vivo*

**FIGURE 9:**
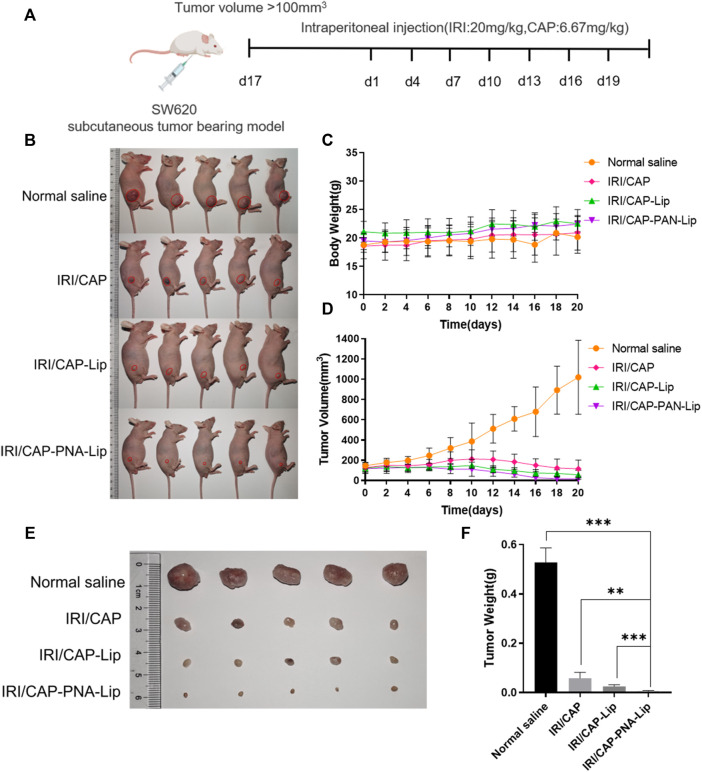
PNA-modified liposomes significantly enhance the antitumor efficacy of the drug. **(A)** Design principles for the analysis of the antitumor abilities of PNA-modified liposomes *in vivo*. **(B)** Photos of tumor-bearing nude mice in each group after 20 days of treatment. **(C)** Changes in the bodyweight of the tumor-bearing nude mice. **(D)** Folding line graph of the change in tumor volume. **(E)** Photographs of tumors. **(F)** Histograms of tumor weights. The data are presented as the mean ± SD (n = 5). **p < 0.01. ***p < 0.001.

Four groups of tumor-bearing nude mice were treated with physiological saline, IRI/CAP, IRI/CAP-Lips, or IRI/CAP-PNA-Lips to evaluate the antitumor ability of PNA-modified liposomes coloaded with IRICAP *in vivo*. There was no significant variation in the bodyweight of mice in the four groups after treatment (Figure 9C). All three dosing groups exhibited antitumor properties, but the tumor volume and weight of the IRI/CAP-PNA-Lip group were the lowest and were even lower than those of the IRI/CAP-Lip group (Figures 9B,D). The results showed that the IRI/CAP-PNA-Lip group had the strongest antitumor effect, with the tumor inhibition rate (TIR) reaching 12.69%, which was 9.6-fold better than that of the free drug group (Figures 9E,F).

### Systemic Toxicity of PNA-Modified Liposomes Coloaded With IRICAP *In Vivo*

**FIGURE 10:**
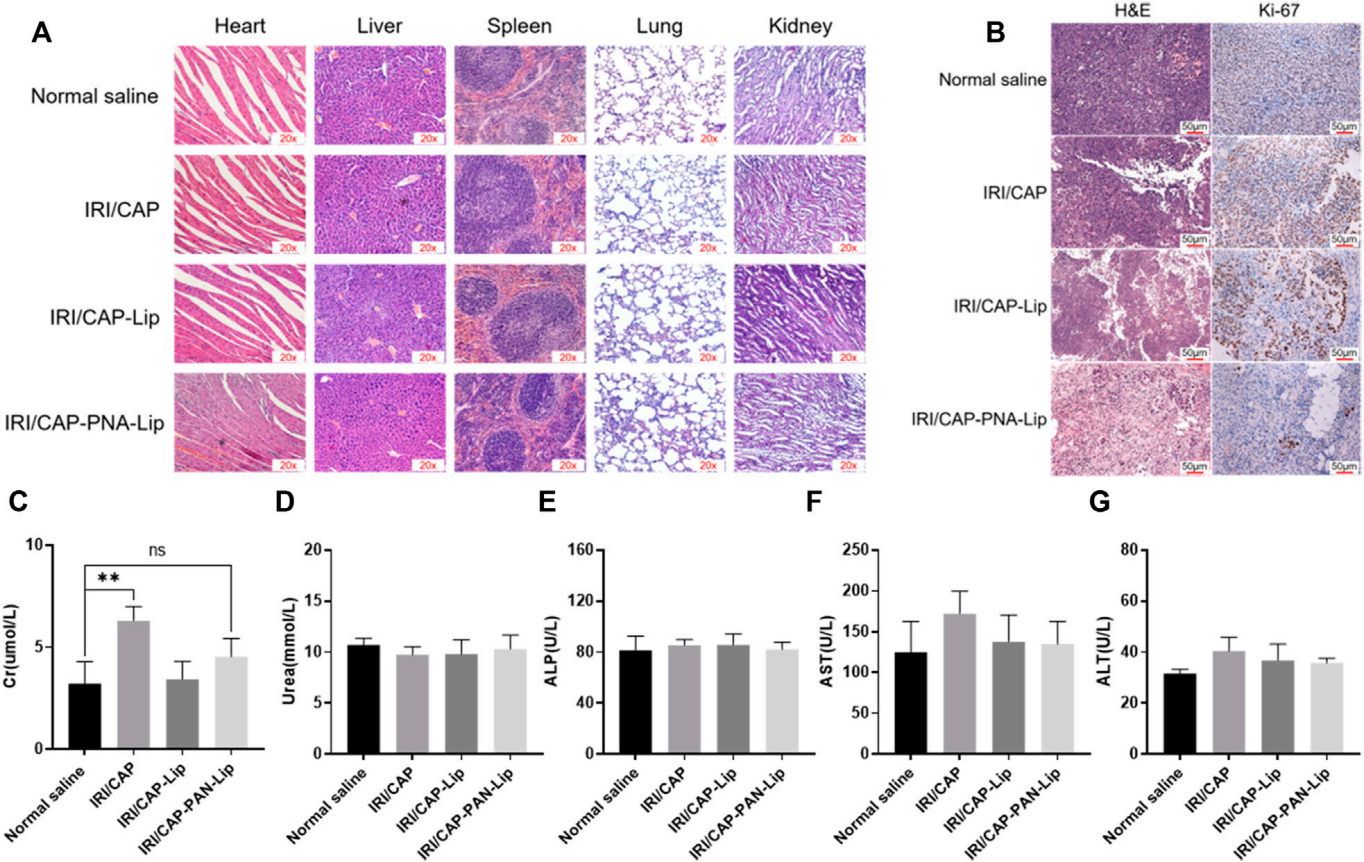
PNA-modified liposomes had no systemic toxicity and enhanced the antiproliferative ability of drugs on MUC1-positive tumors. **(A)** H&E staining of vital organs. No significant pathological changes were observed in any group except for the tumor tissue. 20x **(B)** Ki-67 staining of tumors in different treatment groups. The plasma biochemical analysis of the tumor-bearing nude mice showed no significant difference compared with the saline group except for Cr. **(C)** Creatinine, Cr. **(D)** Urea. **(E)** Alkaline phosphatase, ALP. **(F)** Aspartate aminotransferase, AST. **(G)** Alanine aminotransferase, ALT. **p < 0.01.

To evaluate the systemic toxicity of IRI/CAP-PNA-Lips *in vivo*, H&E staining was performed on the heart, liver, spleen, lung, and kidney. No histopathological abnormalities were observed in the four groups (Figure 10A). The tumor tissues of the saline group were closely arranged with large nuclei, while the tumor tissues of the IRI/CAP-PNA-Lip group presented the highest degree of apoptosis and necrosis, reduced cytokinesis, agglutination of chromatin in the nuclei, and a large number of vacuoles. Immunohistochemistry results showed that Ki-67 expression was decreased in the tumor tissues of the IRI/CAP-PNA-Lip group compared to the IRI/CAP-Lip group, which suggested that PNA-modified liposomes coloaded with IRICAP had a stronger antitumor cell proliferative effect (Figure 10B).

Plasma biochemical analyses were used to assess the effects of PNA-modified liposomes coloaded with IRICAP on the major metabolic organs of tumor-bearing mice. The results showed that except for the creatinine (Cr) value in the free drug group, which was slightly higher than that in the saline group (*p* < 0.01), the biochemical indices of urea, alanine aminotransferase (ALT), alkaline phosphatase (ALP), and aspartate aminotransferase (AST) showed no significant variation. The aforementioned results suggest that PNA-modified liposomes coloaded with IRICAP do not produce any systemic toxicity in the experimental mice (Figures 10C–G).

## Discussion

The desired synergistic effect relies on well-controlled drug dose matching and duration of effect. Peng et al. (2021) used liposomes to codeliver adriamycin and sorafenib, and the results showed that liposomes could achieve codelivery of the drugs and effectively improve the synergistic effect of the drugs. Although coloaded liposomes could improve drug efficacy, they are not specific for tumor tissue. Therefore, ligand-modified liposomes for active targeted drug delivery to enhance antitumor ability can be synthesized. IRI and CAP are synergistic chemotherapies approved by the FDA for treating colorectal cancer, but ligand-modified liposomes coloaded with IRICAP have not been reported (Chiorean et al., 2020). In this study, PNA-modified liposomes coloaded with IRICAP were prepared to specifically and actively target MUC1-positive colorectal cancer tumor tissue to further improve the synergistic efficacy. This drug delivery system can improve drug efficacy and reduce drug dosage while reducing drug concentrations in normal tissues and toxic side effects (Caliskan et al., 2019).

Taking into account the different water solubilities of CAP and IRI, they were encapsulated in hydrophobic or hydrophilic phases. This construction contributed to protecting the chemical stability of the two drugs during preparation, preservation, and blood transport while facilitating the stability of coloaded liposomes (Huang et al., 2019). Nanomedicines with particle size less than 200 nm can accumulate in tumor tissues through the enhanced permeability and retention effect (EPR). In this study, the particle size of each type of liposome fluctuated within approximately 120–130 nm, which is also close to the range reported in other studies (Kalyane et al., 2019; Park et al., 2019; Tan et al., 2019). The PDI of drug-loaded liposomes was between 0.1 and 0.3 in most studies, and the PDI of the liposomes in this study ranged between 0.12 and 0.18 (<0.2), indicating a good dispersion of the liposomes (Yang et al., 2015; Li et al., 2017). To enhance the efficacy of coloaded liposomes, we prepared negative potential liposomes that can protect liposomes from the reticuloendothelial system (RES) and effectively improve the circulation time in the blood (Ghosh et al., 2021). In addition, we hoped that the coloaded liposomes will accumulate and release as much of the drug as possible in the tumor tissue during circulation in blood. The 24-h maximum cumulative drug release of IRI and CAP in IRI/CAP-PNA-Lips was less than 60%, and sustained drug release was successfully achieved compared to the free drug *in vitro*, which reduced unnecessary drug release during circulation in blood (Allen and Cullis, 2013; Yalcin et al., 2018).

By Western blotting, we found that the molecular weight of MUC1 in HT29 and SW620 cells was not consistent, which was consistent with other studies showing that the different degrees of extracellular glycosylation of MUC1 cause the molecular weight to range from 240 to 500 kDa (Nath and Mukherjee, 2014; Apostolopoulos et al., 2015). Subsequently, cellular uptake experiments showed that the coloaded liposomes achieved same-time and same-place drug delivery, which was beneficial in improving the synergistic drug efficacy (Zhang et al., 2017; Du et al., 2020). Meanwhile, the fluorescence signals of FITC/Rhb-PNA-Lips were significantly higher than those of the FITC/Rhb-Lip group due to the specific binding of PNA-modified liposomes to MUC1, which enhanced the cellular uptake of liposomes, and the *in vivo* targeting analyses were consistent with the results *in vitro* (Tang et al., 2020). When we further analyzed the ability of PNA-modified liposomes coloaded with IRICAP to inhibit cell viability *in vitro*, interestingly there was no significant difference in MUC1-positive cells in the IRI/CAP-PNA-Lip group compared to the IRI/CAP-Lip group, which has also been reported in other studies (Zhang et al., 2018). Based on the analysis of the cellular uptake results, we reduced the drug treatment time to 24 h after each group, and IRI/CAP-PNA-Lips showed significant cell viability inhibition compared to the control group. The IRI/CAP-PNA-Lip group showed excellent results in the antitumor assay *in vivo*, with a continuous decrease in tumor volume with a tendency to completely disappear. As a result, we had to euthanize the nude mice no longer than 20 days after treatment. Combined with our previous studies, we suggest that the potential antitumor mechanism of IRI/CAP-PNA-Lips is that colorectal cancer cells improve their uptake of liposomes through receptor-mediated endocytosis, promoting drug entry into the nucleus and inhibiting DNA replication and synthesis, which specifically blocks the S phase of the cell cycle, thereby inhibiting the proliferation of colorectal cancer cells (Li et al., 2020).

There are some limitations of IRI/CAP-PNA-Lips in this study. CAP can be classified as both a water-soluble and a fat-soluble drug, which leads to a lower encapsulation rate in the hydrophobic phase compared to other studies. In this study, we were unable to fully mimic the dose and dosing cycle of IRI in combination with CAP as in clinical treatment. The administration was through intraperitoneal injection for the *in vivo* experiments, and although other studies have shown that small molecules with particle size less than 300 nm can be absorbed through the intestine and IRI/CAP-PNA-Lips exert excellent antitumor effects, there is still a gap in comparison with the effectiveness of clinical intravenous drug administration (Eloy et al., 2016; Kopeckova et al., 2019).

In summary, well-characterized PNA-modified liposomes loaded with IRICAP (IRI/CAP-PNA-Lips) were successfully prepared to achieve synergistic drug delivery and enhanced the synergistic treatment effect in this study. The local drug concentrations and retention times in tumor tissues are increased through targeting ability that further enhances the antitumor effect of the drugs. Both *in vitro* and *in vivo* experiments are suggestive of the excellent antitumor effect of IRI/CAP-PNA-Lips. Therefore, this study is important for optimizing clinical combination treatment protocols, improving the antitumor effect of drugs, and reducing the drug delivery concentration required to achieve beneficial tumor treatment effects. PNA-modified liposomes could be a potential strategy for the treatment of colorectal cancer.

## Data Availability

The original contributions presented in the study are included in the article/Supplementary Material; further inquiries can be directed to the corresponding authors.
