# Supplementary figures and images for: PNA-Modified Liposomes Improve the Delivery Efficacy of CAPIRI for the Synergistic Treatment of Colorectal Cancer
Source: Front Pharmacol. 2022 Jun 15;13:893151. doi: 10.3389/fphar.2022.893151 (PMC9240350; doi:10.3389/fphar.2022.893151)

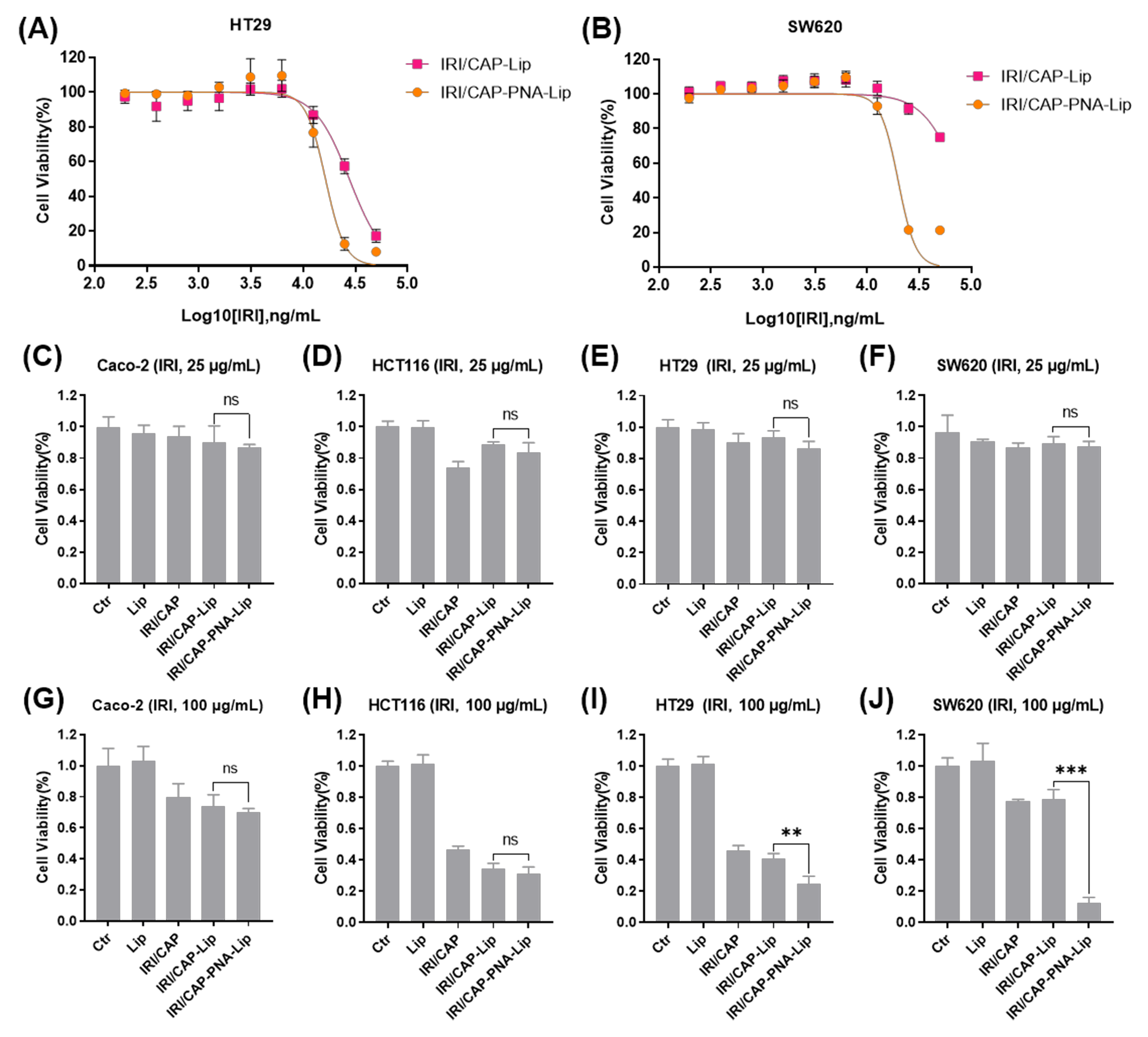

Supplement: Supplementary file 1 [file Image3.TIF]

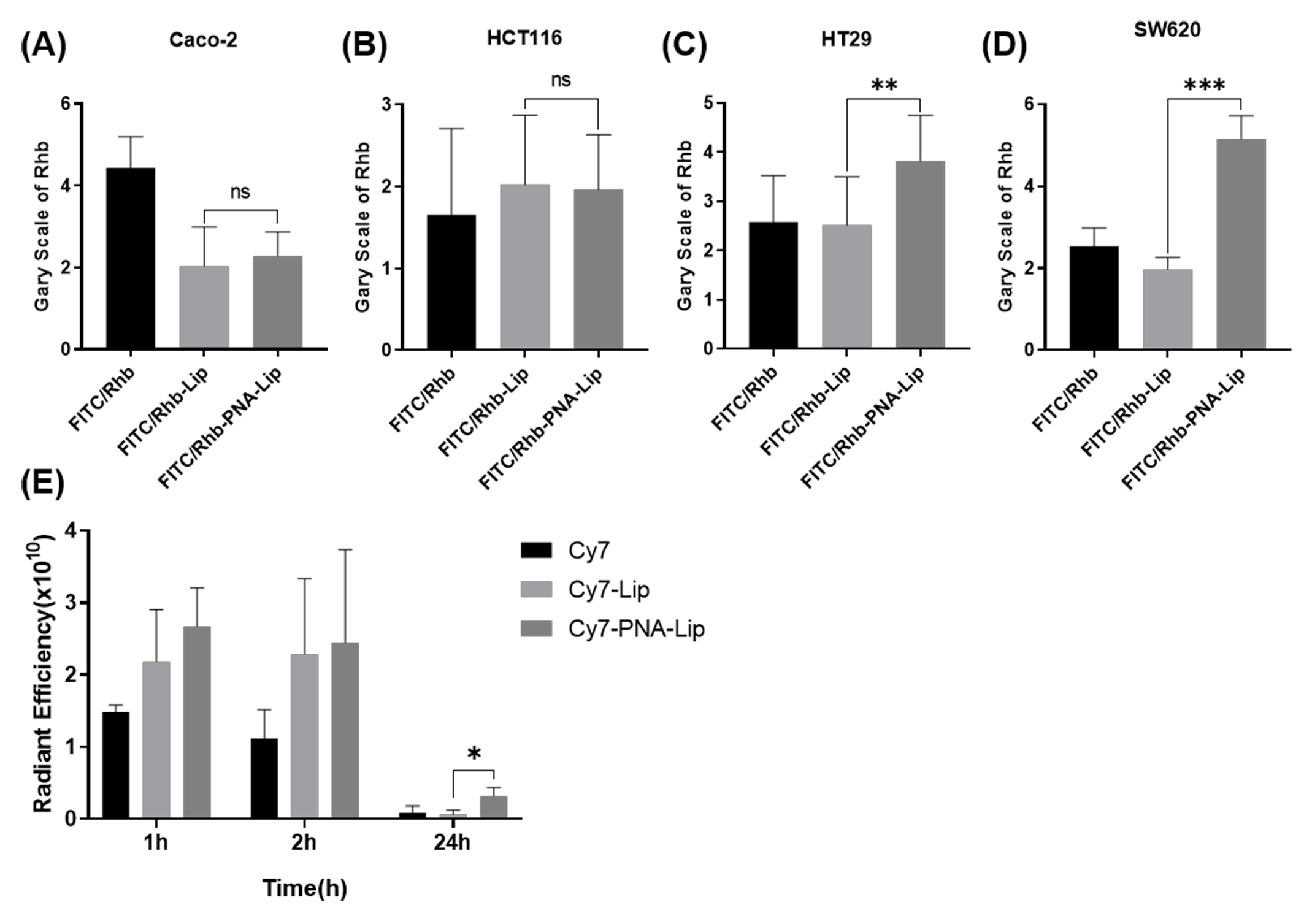

Supplement: Supplementary file 2 [file Image2.TIF]

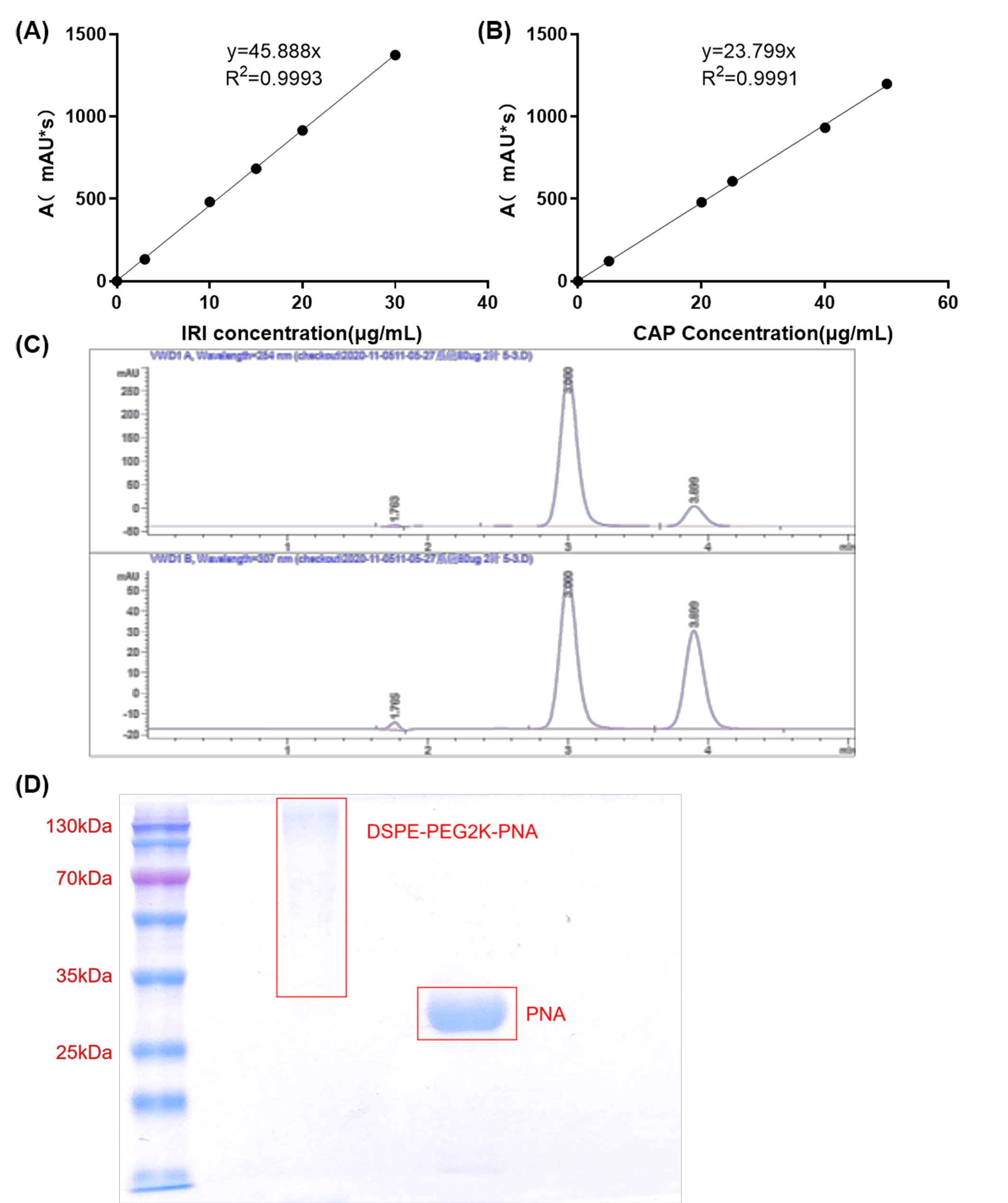

Supplement: Supplementary file 3 [file Image1.TIF]
